# IFN-γ-inducible protein of 10 kDa upregulates the effector functions of eosinophils through β_2 _integrin and CXCR3

**DOI:** 10.1186/1465-9921-12-138

**Published:** 2011-10-17

**Authors:** Yotaro Takaku, Kazuyuki Nakagome, Takehito Kobayashi, Koichi Hagiwara, Minoru Kanazawa, Makoto Nagata

**Affiliations:** 1 Department of Respiratory Medicine, Saitama Medical University, 38 Morohongo, Moroyama-cho, Iruma-gun, Saitama, Japan; 2 Allergy Center, Saitama Medical University, 38 Morohongo, Moroyama-cho, Iruma-gun, Saitama, Japan

**Keywords:** asthma, acute exacerbation, ICAM-1, IP-10, rhinovirus

## Abstract

**Background:**

Eosinophils play an important role in the pathogenesis of bronchial asthma and its exacerbation. Recent reports suggest the involvement of IFN-γ-inducible protein of 10 kDa (IP-10) in virus-induced asthma exacerbation. The objective of this study was to examine whether CXCR3 ligands including IP-10 modify the effector functions of eosinophils.

**Methods:**

Eosinophils isolated from the blood of healthy donors were stimulated with CXCR3 ligands and their adhesion to rh-ICAM-1 was then measured using eosinophil peroxidase assays. The generation of eosinophil superoxide anion (O_2_^-^) was examined based on the superoxide dismutase-inhibitable reduction of cytochrome C. Eosinophil-derived neurotoxin (EDN) release was evaluated to determine whether CXCR3 ligands induced eosinophil degranulation. Cytokine and chemokine production by eosinophils was examined using a Bio-plex assay.

**Results:**

Eosinophil adhesion to ICAM-1 was significantly enhanced by IP-10, which also significantly induced eosinophil O_2_^- ^generation in the presence of ICAM-1. Both the enhanced adhesion and O_2_^- ^generation were inhibited by an anti-β_2 _integrin mAb or an anti-CXCR3 mAb. Other CXCR3 ligands, such as monokine induced by IFN-γ (Mig) and IFN-inducible T cell α chemoattractant (I-TAC), also induced eosinophil adhesion and O_2_^- ^generation in the presence of ICAM-1. IP-10, but not Mig or I-TAC, increased the release of EDN. IP-10 increased the production of a number of cytokines and chemokines by eosinophils.

**Conclusions:**

These findings suggest that CXCR3 ligands such as IP-10 can directly upregulate the effector functions of eosinophils. These effects might be involved in the activation and infiltration of eosinophils in the airway of asthma, especially in virus-induced asthma exacerbation.

## Background

Bronchial asthma is a chronic disorder characterized by airway inflammation, reversible airway obstruction, mucus hypersecretion, and airway hyperresponsiveness [1]. A variety of cells, including eosinophils, T lymphocytes, mast cells, neutrophils, and dendritic cells, are involved in the process of airway inflammation of asthma. Of these cells, eosinophils preferentially accumulate at sites of allergic inflammation and are believed to play important roles in the pathophysiology of asthma through the release of a variety of inflammatory mediators, including major basic protein, cysteinyl leukotrienes (cysLTs), reactive oxygen species and cytokines [2,3]. Green *et al. *reported that a treatment strategy directed at normalization of the induced sputum eosinophil count reduces asthma exacerbations [4], suggesting an important role for eosinophils in the pathogenesis of asthma exacerbation.

Acute respiratory infections are a major cause of asthma exacerbation [5,6]. Clinical data suggest that not only the numbers of neutrophils, but also that of eosinophils increases in asthmatic airways during, or following, viral infection [7-9]. For example, experimental viral infection increases eosinophil counts in the epithelium of patients with allergic asthma [7]. The sputum of asthmatic patients with confirmed viral infection contains high levels of eosinophilic cationic protein [8]. These findings suggest that eosinophils are indeed activated and infiltrate asthmatic airways during or following viral infection.

The initial steps of eosinophil accumulation in the asthmatic airway are adhesion to and subsequent migration across endothelial cells. Eosinophil adhesion molecules, such as α_4 _integrins including α_4_β_1 _(VLA-4, CD49d/CD29) and β_2 _integrins including α_L_β_2 _(LFA-1, CD11a/CD18) and α_M_β_2 _(Mac-1, CD11b/CD18), play roles in the adhesion to and transmigration through the endothelium [10]. Furthermore, eosinophil adhesion to adhesion molecules, such as vascular cell adhesion molecule (VCAM)-1 (a ligand for α_4 _integrins) and intercellular adhesion molecule (ICAM)-1 (a ligand for β_2 _integrins) may augment eosinophil function [11-13]. For example, the adhesion of eosinophils to VCAM-1 stimulates eosinophil superoxide anion (O_2_^-^) generation [11]. Similarly, adhesion of eosinophils to ICAM-1 augments both leukotriene (LT) C_4 _generation and the release of eosinophil-derived neurotoxin (EDN) [12]. Therefore, interaction between eosinophils and adhesion molecules would contribute to the development of airway inflammation in bronchial asthma.

IFN-γ-inducible protein of 10 kDa (IP-10) belongs to the CXC chemokine subfamily that binds to the common receptor, CXCR3, and it is produced by various cell types in response to IFN-γ [14]. CXCR3 is expressed at high levels on Th0 and Th1 lymphocytes. Jinquan *et al. *reported that CXCR3 is also expressed on eosinophils [15]. Recently, the role of IP-10 in virus-induced asthma exacerbation has been highlighted [16-18]. However, whether IP-10 actually modifies allergic airway inflammation, including the accumulation or activation of eosinophils, has not been fully investigated.

Monokine induced by IFN-γ (Mig) and IFN-inducible T cell α chemoattractant (I-TAC) are also CXC chemokines [19]. However, the roles of Mig or I-TAC in the pathogenesis of asthma and their effects on eosinophils have not been elucidated in detail.

Here, we examined the effect of the CXCR3 ligands IP-10, Mig and I-TAC on eosinophil functions such as their adhesion properties, O_2_^- ^generation, EDN release and cytokine production, in the presence or absence of adhesion molecules such as ICAM-1.

## Methods

### Preparation of eosinophils

Eosinophils were isolated from peripheral blood specimens collected from non-atopic healthy donors with a peripheral blood differential eosinophil count of < 5%. The numbers of males and females, ranging in age from 24 to 42 years, were comparable among the donors. Written, informed consent was obtained from each donor before collecting blood samples. Eosinophils were isolated by negative selection using immunomagnetic beads as described [11-13,20-23]. Over 98% of the cells were eosinophils, as determined by morphologic criteria using May-Grünwald-Giemsa staining. We also confirmed by flow cytometry that over 98% of the cells were CD16-negative/CD14-negative (data not shown). Eosinophil viability was > 99%, as determined by Trypan blue dye exclusion. Eosinophils were resuspended in Hank's balanced salt solution (HBSS) supplemented with gelatin to a final concentration of 0.1% (HBSS/gel).

### Eosinophil adhesion assay

Eosinophil adhesion to rh-ICAM-1- or rh-VCAM-1-coated plates was assessed based on the residual eosinophil peroxidase (EPO) activity of adherent eosinophils as described [11-13,20]. Eosinophils (100 μl of 1 × 10^5 ^cells/ml in HBSS/gel) were incubated at 37°C for 20 min in rh-ICAM-1- or rh-VCAM-1-coated plates in the presence or absence of the CXCR3 ligands IP-10 (R&D Systems, Minneapolis, MN), Mig (R&D Systems), or I-TAC (R&D Systems). In selected experiments, eosinophils were incubated with anti-α_4_-integrin mAb (3 μg/ml; clone HP1/2; Cosmo Bio Co. Ltd, Tokyo, Japan), anti-β_2_-integrin mAb (3 μg/ml; clone L130; Becton Dickinson, Franklin Lakes, NJ), or anti-CXCR3 mAb (3 μg/ml; clone 49801; R&D Systems, Minneapolis, MN) before assay. The plates were washed with HBSS and 100 μl of HBSS/fetal calf serum (FCS) was then added to the wells. Standards comprised of 100 μl of serially diluted cell suspensions (1 × 10^3^, 3 × 10^3^, 1 × 10^4^, 3 × 10^4^, and 1 × 10^5 ^cells/ml) were added to empty wells. The EPO substrate (1 mM *o*-phenylenediamine, 1 mM H_2_O_2_, and 0.1% Triton X-100 in Tris buffer, pH 8.0) was then added to all wells and the plates were incubated for 30 min at room temperature. The reaction was stopped by adding 50 μl of 4 M H_2_SO_4 _and absorbance was measured at 490 nm. Each experiment was performed in quadruplicate using eosinophils from a single donor, and percentage eosinophil adhesion was determined from mean values that were calculated from log dose-response curves. Eosinophil viability after incubation was > 98%, as determined by Trypan blue dye exclusion.

### Eosinophil superoxide anion generation

Eosinophil O_2_^- ^generation was measured in 96-well plates (Corning Inc.) as described based on the superoxide dismutase (SOD)-inhibitable reduction of cytochrome C [11-13]. We initially added SOD (0.2 mg/ml in HBSS/gel; 20 μl) to SOD control wells and then HBSS/gel to all wells to bring the final volume to 100 μl. The eosinophil density was adjusted to 1.25 × 10^6 ^cells/ml of HBSS/gel mixed 4:1 with cytochrome C (12 mg/ml of HBSS/gel), and 100 μl of eosinophil suspension was then added to all wells. Immediately after adding CXCR3 ligand, the absorbance of the cell suspensions in the wells was measured at 550 nm in an Immuno-Mini (NJ-2300; Japan Intermed Co., Tokyo, Japan), followed by repeated measurements over the next 240 min. In selected experiments, eosinophils were incubated with anti-β_2_-integrin mAb (3 μg/ml) or anti-CXCR3 mAb (3 μg/ml) before assay. The plates were incubated in a 5% CO_2 _incubator at 37°C between measurements. Each reaction was evaluated in duplicate against the control reaction in wells containing 20 μg/ml of SOD. The results were adjusted for a 1-ml reaction volume, and O_2_^- ^generation was calculated at an extinction coefficient of 21.1 mM^-1^cm^-1^, as nanomoles of cytochrome C reduced per 1.0 × 10^6 ^cells/ml minus the SOD control [9-11]. The maximum value during the incubation time was examined to evaluate the effects of various factors on eosinophil O_2_^- ^generation. Cell viability, determined by Trypan blue exclusion at the end of each experiment, remained at 95% after a 240-min incubation with the activator.

### Eosinophil Degranulation

Eosinophils (1 × 10^6 ^cells/ml) in 96-well plates were incubated for the 240 min that were required for measurement of O_2_^- ^generation, and were then immediately centrifuged (700 g) at 4°C for 15 min. Recovered cell-free supernatants were subjected to EDN analysis, as described previously [21]. Levels of EDN were quantified using ELISA kits (Medical and Biological Laboratory Co Ltd, Nagoya, Japan).

### Cytokine and chemokine production

Eosinophils (1 × 10^6 ^cells/ml) were resuspended in RPMI 1640 containing 10% FCS. Cells were incubated with CXCR3 ligands in 96-well tissue-culture plates for 24 h at 37°C and 5% CO_2 _[24]. After culture, the supernatants were collected, and cytokine and chemokine concentrations were measured by using Bio-plex assay kits (Bio-Rad, Mississauga, Canada).

### Statistical analysis

Values are expressed as means ± SEM. Two groups were compared using Student's *t *test and more than two groups were compared using a repeated-measures analysis of variance (ANOVA) with Tukey's test of multiple comparisons. Values of *P *< 0.05 were considered statistically significant.

## Results

### Effect of IP-10 on eosinophil adhesiveness

**Figure 1:**
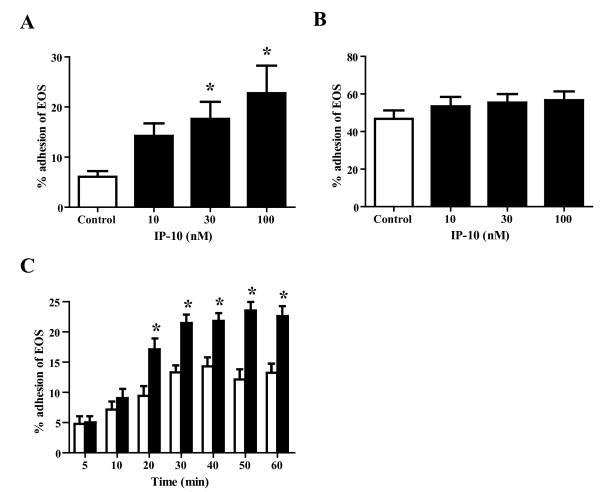
**IP-10 significantly enhances the adhesiveness of eosinophils**. (A) IP-10 significantly augments eosinophil adhesion to rh-ICAM-1-coated plates. Eosinophils (100 μl of 1 × 10^5 ^cells/ml in HBSS/gel) obtained from different healthy donors, were incubated with IP-10 (10-100 nM) at 37°C for 20 min in rh-ICAM-1-coated plates. The adhesiveness of the eosinophils was then assessed by assay of residual EPO activity. Data are shown as means ± SEM of 5 experiments using cells from different donors. (B) IP-10 does not modify eosinophil adhesion to rh-VCAM-1-coated plates. Eosinophils were incubated with IP-10 (10-100 nM) in rh-VCAM-1-coated plates, and the adhesiveness of the eosinophils was examined. Data are shown as means ± SEM of 5 experiments using cells from different donors. (C) Time course of IP-10 induction of eosinophil adhesion to ICAM-1 coated plates. Eosinophils were incubated with or without IP-10 (100 nM) for periods ranging from 5 to 60 min. The kinetics of eosinophil adhesion to ICAM-1 coated plates was determined by aspiration of non-adhered cells at the indicated times, followed by washing every 5 min for the next 10 min and then every 10 min over the next 50 min, following which adhesion was assayed. Data are shown as means ± SEM of 6 experiments using cells from different donors. Open and filled bars, eosinophil adhesion without and with IP-10, respectively. *P < 0.05 versus without IP-10.

We initially examined whether or not IP-10 directly modifies eosinophil adhesiveness. We found that IP-10 (100 nM) significantly enhanced the adhesiveness of eosinophils to plastic plates compared with controls (data not shown), suggesting that IP-10 modifies adhesive interactions between eosinophils and adhesion molecules. To test this hypothesis, we investigated the effect of IP-10 on eosinophil adhesion to recombinant adhesion molecules. Eosinophils were stimulated with rh-IP-10 (10-100 nM) in the presence of immobilized rh-ICAM-1 or rh-VCAM-1, and adhesion was then evaluated. Under these conditions, IP-10 did not significantly increase eosinophil adhesion to ICAM-1 at a concentration of 10 nM, but did significantly enhance adhesion to ICAM-1 at a concentration of 30 and 100 nM, compared with the control (14.3 ± 2.5%, 17.6 ± 3.4% and 22.8 ± 5.5% vs. 6.1 ± 1.1%; NS, *P *< 0.05 and *P *< 0.05, respectively; n = 5 each; Figure 1A). In contrast, IP-10 (100 nM) did not enhance eosinophil adhesion to either VCAM-1 or fibronectin, which are counter ligands for eosinophil α_4 _integrin, compared with controls (46.8 ± 4.5% vs. 56.7 ± 4.6% and 20.4 ± 2.8% vs. 20.2 ± 1.8%, respectively; both NS; n = 5 each; Figure 1B and data not shown). We evaluated the effects of incubation with IP-10 on the adhesiveness of eosinophils to ICAM-1 over time. Eosinophils were incubated with or without IP-10 (100 nM) for periods ranging from 5 to 60 min, washed, and then adhesion to ICAM-1 was evaluated. The results showed that IP-10 (100 nM) augmented eosinophil adhesiveness started from 20 min and lasted for 60 min (*P *< 0.05 for all time points between 20 and 60 min, n = 6; Figure 1C).

### Effects of anti-integrin mAbs on eosinophil adhesion enhanced by ICAM-1 and IP-10

**Figure 2:**
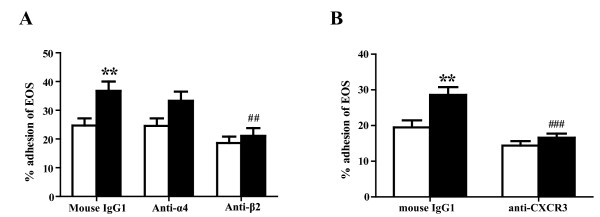
**Anti-β**_**2 **_**integrin mAb and anti-CXCR3 mAb suppress eosinophil adhesion enhanced by ICAM-1 and IP-10**. (A) IP-10-enhanced eosinophil adhesion to ICAM-1 is inhibited by anti-β_2 _integrin mAb. Eosinophils were pre-incubated with either anti-integrin mAb (3 μg/ml) or control IgG for 15 min prior to analysis of adhesion in the presence or absence of IP-10. Data are shown as means ± SEM of 6 experiments using cells from different donors. (B) IP-10-enhanced eosinophil adhesion to ICAM-1 is inhibited by anti-CXCR3 mAb. Eosinophils were pre-incubated with either anti-CXCR3 mAb (3 μg/ml) or control IgG for 15 min prior to analysis of adhesion in the presence or absence of IP-10. Data are shown as means ± SEM of six experiments using cells from different donors. Open and filled bars, eosinophil adhesion without and with IP-10, respectively. **P < 0.01 versus without IP-10. ^##^*P *< 0.01, ^###^*P *< 0.001 versus mouse IgG1.

To identify the eosinophil integrin(s) involved in IP-10-induced eosinophil adhesion to ICAM-1, we incubated eosinophils with anti-β_2 _integrin mAb, anti-α_4 _integrin mAb, or an isotype-matched control mouse IgG1 at ambient temperature for 15 min, and then evaluated adhesion. Eosinophil adhesion that was enhanced by ICAM-1 plus IP-10 was inhibited by anti-β_2 _integrin mAb (mouse IgG1 vs. anti-β_2 _integrin mAb: 36.8 ± 3.2% vs. 21.1 ± 2.7%, *P *< 0.01; n = 6 each; Figure 2A). Anti-α_4 _integrin mAb did not modify the ICAM-1 and IP-10-enhanced eosinophil adhesion (mouse IgG1 vs. anti-α_4 _integrin mAb: 36.8 ± 3.2% vs. 33.3 ± 3.2%, NS; n = 6 each; Figure 2A). Neither anti-α_4 _integrin mAb nor anti-β_2 _integrin mAb modified spontaneous eosinophil adhesion (Figure 2A).

### Effect of anti-CXCR3 mAb on eosinophil adhesion enhanced by ICAM-1 and IP-10

We examined whether CXCR3, a major receptor for CXC chemokines, is involved in IP-10-induced eosinophil adhesion to ICAM-1. Eosinophils were incubated with either anti-CXCR3 mAb or an isotype-matched control mouse IgG1 at ambient temperature for 15 min before adhesion assays. Anti-CXCR3 mAb inhibited the eosinophil adhesion that was enhanced by ICAM-1 plus IP-10 (mouse IgG1 vs. anti-CXCR3 mAb: 28.5 ± 2.2 vs. 16.5 ± 1.2%; *P *< 0.001; n = 6; Figure 2B), suggesting that IP-10 could directly upregulate eosinophil adhesion via CXCR3.

### Effect of IP-10 on eosinophil O_2_^- ^generation in the presence of ICAM-1

**Figure 3:**
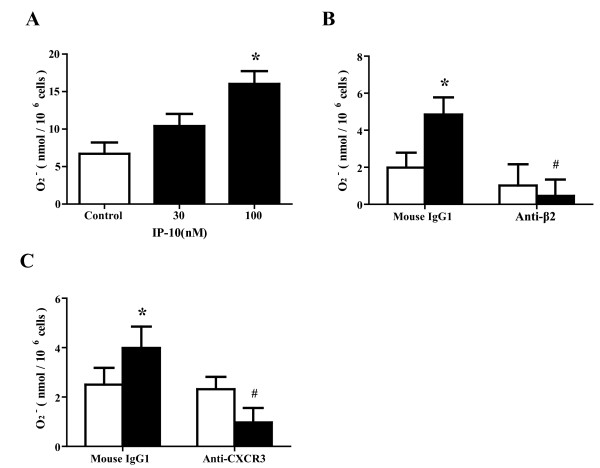
**IP-10 significantly enhances eosinophil O**_**2**_^- ^**generation**. (A) IP-10 significantly enhances eosinophil O_2_^- ^generation in the presence of ICAM-1. Eosinophil cell density was adjusted to 1.25 × 10^6 ^cells/ml of HBSS/gel mixed 4:1 with cytochrome C, and the eosinophil suspension was then added to ICAM-1 coated 96-well plates. Immediately after adding IP-10 (30 or 100 nM), eosinophil O_2_^- ^generation was measured based on the SOD-inhibitable reduction of cytochrome C. Data are shown as means ± SEM of 5 experiments using cells from different donors. (B) Eosinophil O_2_^- ^generation induced by the combination of IP-10 + ICAM-1 is blocked by anti-β_2 _integrin mAb. Eosinophils were pre-incubated with anti-β_2 _integrin mAb (3 μg/ml) or control IgG for 15 min prior to analysis of O_2_^- ^generation of eosinophils as in (A). Data are shown as means ± SEM of 7 experiments using cells from different donors. (C) Eosinophil O_2_^- ^generation induced by a combination of IP-10 + ICAM-1 is blocked by anti-CXCR3 mAb. Eosinophils were pre-incubated with anti-CXCR3 mAb (3 μg/ml) or control IgG for 15 min prior to analysis of O_2_^- ^generation of eosinophils as in (A). Data are shown as means ± SEM of 7 experiments using cells from different donors. Open and filled bars, eosinophil O_2_^- ^generation without and with IP-10, respectively. *P < 0.05 versus without IP-10. ^#^*P *< 0.05 versus mouse IgG1.

We next examined whether IP-10 modifies O_2_^- ^generation of eosinophils in the presence of ICAM-1. IP-10 did not affect O_2_^- ^generation by eosinophils at a concentration of 30 nM (Figure 3A). However, at a concentration of 100 nM, IP-10 significantly activated eosinophil O_2_^- ^generation in wells coated with ICAM-1 compared with controls (5.2 ± 1.8 vs. 8.5 ± 3.0 nmol/10^6 ^cells, *P *< 0.05; n = 6; Figure 3A), although the effect of IP-10 on eosinophil O_2_^- ^generation was of small magnitude.

### Effect of anti-β_2 _integrin or anti-CXCR3 mAb on eosinophil O_2_^- ^generation induced by a combination of ICAM-1 and IP-10

We investigated whether β_2_integrin or CXCR3 is involved in the enhanced eosinophil O_2_^- ^generation induced by ICAM-1 plus IP-10, by assay of the effect of pre-incubating eosinophils with anti-β_2 _integrin mAb, anti-CXCR3 mAb, or an isotype-matched control mouse IgG1 at ambient temperature for 15 min prior to analysis. Eosinophil O_2_^- ^generation induced by the combination of ICAM-1 and IP-10 was blocked by both anti-β_2 _integrin and anti-CXCR3 mAbs compared with mouse IgG1 (4.9 ± 0.9 vs. 0.5 ± 0.9 and 4.0 ± 0.9 vs. 1.0 ± 0.6 nmol/10^6 ^cells, respectively; n = 7 each; *P *< 0.05 for both; Figures 3B and 3C). Neither anti-β_2 _integrin mAb nor anti-CXCR3 mAb modified spontaneous O_2_^- ^generation by eosinophils.

### Effects of other CXCR3 ligands on adhesion and O_2_^- ^generation of eosinophils

**Figure 4:**
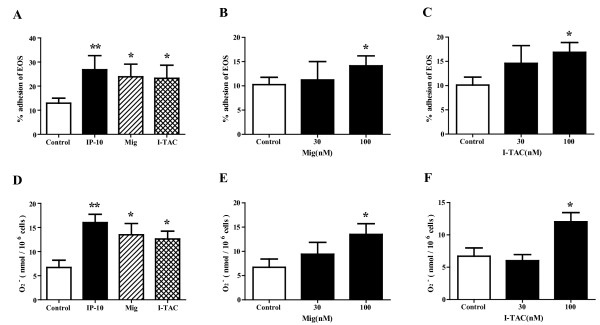
**Other CXCR3 ligands also enhance eosinophil adhesion and eosinophil O**_**2**_^- ^**generation**. (A-C) All tested CXCR3 ligands significantly augmented eosinophil adhesion to ICAM-1-coated plates. Eosinophils were incubated with or without CXCR3 ligands for 20 min, and adhesion of eosinophils was then examined. (A) All of the CXCR3 ligands (100 nM) augmented eosinophil adhesion to ICAM-1. (B) Dose-response relationship between Mig and eosinophil adhesion. (C) Dose-response relationship between I-TAC and eosinophil adhesion. Data shown are means ± SEM of 5 experiments using cells from different donors. (D-F) All tested CXCR3 ligands significantly induced eosinophil O_2_^- ^generation in the presence of ICAM-1. Eosinophil O_2_^- ^generation was measured with or without CXCR3 ligands. (D) All of the CXCR3 ligands (100 nM) induced eosinophil O_2_^- ^generation. (E) Dose-response relationship between Mig and eosinophil O_2_^- ^generation. (F) Dose-response relationship between I-TAC and eosinophil O_2_^- ^generation. Data shown are means ± SEM of 5 experiments using cells from different donors. *P < 0.05, **P < 0.01 versus without CXCR3 ligands.

We examined whether CXCR3 ligands other than IP-10 modify eosinophil adhesion and O_2_^- ^generation by stimulation of eosinophils with IP-10, Mig or I-TAC in the presence of ICAM-1. At a concentration of 100 nM, all of these CXCR3 ligands enhanced eosinophil adhesion to ICAM-1 versus control (IP-10, Mig and I-TAC: 26.8 ± 5.8%, 23.9 ± 5.2% and 23.2 ± 5.4% respectively vs. 12.9 ± 2.1%; *P *< 0.01, *P *< 0.05 and *P *< 0.05; n = 5 each; Figure 4A). However, in contrast to IP-10, 30 nM of Mig or I-TAC did not increase eosinophil adhesion to ICAM-1 (Figures 4B and 4C), suggesting that the effect of Mig or I-TAC on eosinophil adhesion was weaker than that of IP-10. All of the CXCR3 ligands augmented eosinophil O_2_^- ^generation in the presence of ICAM-1 at a concentration of 100 nM, but not at a concentration of 30 nM (IP-10, Mig and I-TAC: 16.0 ± 1.7, 13.5 ± 2.2 and 12.0 ± 1.4, respectively, vs. 6.7 ± 1.5 nmol/10^6 ^cells; *P *< 0.01, *P *< 0.05 and *P *< 0.05; n = 5 each; Figures 4D-F).

### Effect of CXCR3 ligands on EDN release or cytokine/chemokine production by eosinophils

**Figure 5:**
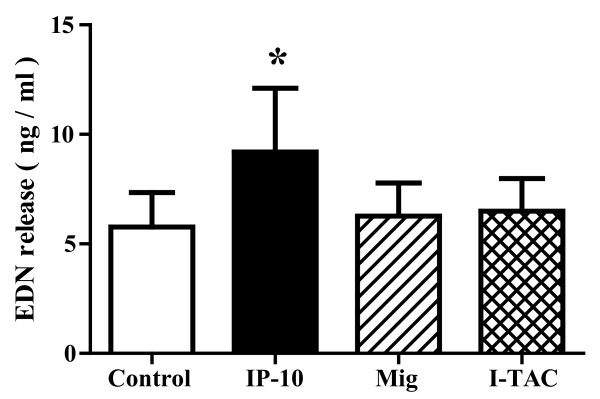
**IP-10, but not Mig or I-TAC, induces eosinophil release of EDN**. Eosinophils (1 × 10^6 ^cells/ml) in 96-well plates were incubated for the 240-min required to measure O_2_^- ^generation, immediately centrifuged and the concentration of EDN in cell-free supernatants was then quantified using ELISA. Data shown are means ± SEM of 6 experiments using cells from different donors. *P < 0.05 versus without CXCR3 ligands.

We examined the effect of CXCR3 ligands on the degranulation of eosinophils as well as the production of cytokines and chemokines by eosinophils. Eosinophils were incubated with CXCR3 ligands and eosinophil degranulation was evaluated by measurement of the concentration of eosinophil-derived neurotoxin (EDN) in the supernatant. At a concentration of 100 nM, IP-10, but not Mig or I-TAC, increased EDN release (IP-10, Mig and I-TAC: 9.2 ± 2.9, 6.3 ± 1.5 and 6.5 ± 1.5, respectively, vs. 5.8 ± 1.6 ng/ml; *P *< 0.05, NS and NS; n = 6 each; Figure 5), suggesting that only IP-10 induced the degranulation of eosinophils. IP-10 did not increase EDN release at a concentration of 30 nM (data not shown). We next measured the concentrations of cytokines and chemokines in supernatant using a Bio-plex assay. As shown in Table 1, IP-10 at a concentration of 100 nM significantly increased the production of several cytokines/chemokines, including proinflammatory cytokines and Th1/Th2 cytokines, by eosinophils. Although Mig or I-TAC increased MIP-1β or IL-9 respectively at a concentration of 100 nM, almost every cytokine/chemokine was not upregulated by stimulation with Mig or I-TAC. To confirm the reliability of the Bio-plex assay kits, we measured IL-6 and IL-8 concentrations by ELISA, and similar results were obtained (data not shown).

**Table 1:** Production of cytokines and chemokines by eosinophils stimulated with CXCR3 ligands.

Cytokine	Control (pg/ml)	IP-10 (pg/ml)	Mig (pg/ml)	I-TAC (pg/ml)
IL-1ra	1.8 ± 0.7	25.0 ± 2.1*	3.8 ± 1.3	3.9 ± 0.9
IL-1β	0.3 ± 0.1	6.5 ± 2.5*	2.6 ± 1.0	1.8 ± 0.9
IL-2	0.0 ± 0.0	2.0 ± 0.1*	0.1 ± 0.1	0.1 ± 0.0
IL-4	0.1 ± 0.0	1.1 ± 0.1*	0.1 ± 0.0	0.1 ± 0.0
IL-5	0.0 ± 0.0	0.3 ± 0.1*	0.0 ± 0.0	0.0 ± 0.0
IL-6	9.2 ± 4.9	80.5 ± 27.4*	40.5 ± 17.4	29.5 ± 14.8
IL-7	0.1 ± 0.0	0.9 ± 0.1*	0.2 ± 0.1	0.2 ± 0.0
IL-8	186.8 ± 89.8	404.7 ± 73.3*	315.3 ± 79.7	251.7 ± 76.5
IL-9	1.1 ± 0.2	4.4 ± 0.4*	1.8 ± 0.3	2.2 ± 0.3*
IL-10	0.7 ± 0.2	2.1 ± 0.4*	1.2 ± 0.4	1.7 ± 0.6
IL-12(p70)	0.3 ± 0.1	0.9 ± 0.1*	0.4 ± 0.1	0.3 ± 0.1
IL-13	1.1 ± 0.3	1.8 ± 0.2*	1.4 ± 0.2	1.4 ± 0.2
IL-15	0.2 ± 0.0	0.5 ± 0.1*	0.3 ± 0.1	0.3 ± 0.1
IL-17	0.8 ± 0.2	2.5 ± 0.7*	0.6 ± 0.2	0.7 ± 0.4
Eotaxin	1.7 ± 0.6	23.2 ± 0.5*	2.4 ± 1.1	2.4 ± 0.9
FGF	1.4 ± 0.5	5.6 ± 1.3*	1.1 ± 0.6	3.1 ± 1.1
G-CSF	0.2 ± 0.1	2.5 ± 0.4*	0.5 ± 0.2	0.3 ± 0.2
GM-CSF	0.8 ± 0.3	9.4 ± 1.0*	1.6 ± 0.4	1.7 ± 0.5
IFN-γ	2.6 ± 0.6	107.0 ± 10.3*	5.4 ± 2.1	5.6 ± 2.0
MCP-1	32.6 ± 14.3	86.5 ± 28.7*	65.5 ± 24.6	53.5 ± 22.1
MIP-1α	6.4 ± 2.8	30.7 ± 10.4*	17.8 ± 8.9	14.5 ± 7.9
MIP-1β	93.7 ± 31.0	183.5 ± 19.1*	160.0 ± 24.5*	135.5 ± 25.7
PDGF	0.6 ± 0.3	2.2 ± 0.5*	0.4 ± 0.1	1.2 ± 0.3
RANTES	6.5 ± 0.9	20.3 ± 1.0*	7.8 ± 1.1	7.9 ± 1.0
TNF-α	4.0 ± 1.9	19.2 ± 4.5*	5.3 ± 3.3	2.7 ± 1.0
VEGF	7.8 ± 2.6	13.3 ± 2.5*	10.5 ± 3.3	10.4 ± 2.8

Eosinophils were incubated with CXCR3 ligands for 24 h, and the concentrations of cytokines and chemokines in cell-free supernatants were measured using Bio-plex assay kits. *P < 0.05 versus without CXCR3 ligands.

## Discussion

We found that IP-10, a CXCR3 ligand, augments eosinophil adhesiveness. This effect occurred when the counter-adhesion protein was ICAM-1, but not VCAM-1. We also found that IP-10 activates eosinophil O_2_^- ^generation in the presence of ICAM-1. Anti-β_2 _integrin or anti-CXCR3 mAb inhibited the induction of eosinophil adhesion or O_2_^- ^generation in the presence of ICAM-1, suggesting that the effect of CXCR3 ligands involves eosinophil β_2 _integrin and CXCR3. The other CXCR3 ligands, Mig and I-TAC, also significantly enhanced eosinophil adhesion to ICAM-1 and induced eosinophil O_2_^- ^generation in the presence of ICAM-1. Furthermore, IP-10 increased the release of EDN and the production of a number of cytokines and chemokines. These results suggest novel roles for CXCR3 ligands including IP-10 as activators of eosinophils through β_2 _integrin and CXCR3.

Although allergic airway inflammation is generally considered to be a process that is mediated by the Th2-type response, recent reports suggest that the Th1-type response also plays an important role. For example, the passive transfer of OVA-specific Th1 cells exacerbates OVA-induced airway eosinophilia in a mouse model [25]. Because IP-10 is originally generated by IFN-γ, and CXCR3 is predominantly expressed on Th1 cells [26], IP-10 is thought to play an important role in the Th1-mediated immune response. However, similar to Th1 cells, IP-10 itself can exacerbate Th2-mediated airway inflammation. For example, IP-10 overexpression in the lung of a mouse model of allergic airway inflammation increases airway hyperreactivity, eosinophilia, and IL-4 levels [27]. Furthermore, IP-10 deletion attenuated Th2-type allergic airway inflammation in a mouse model of asthma [27]. These results demonstrated that the Th1-type chemokine IP-10 rather enhances Th2-mediated eosinophilic inflammation. The mechanism through which IP-10 exacerbates Th2-mediated inflammation remains obscure.

Most asthma exacerbation in a clinical setting is induced by viral respiratory infections, in particular those caused by the rhinovirus (RV) [5]. Although eosinophils, as well as neutrophils, are increased in asthmatic airways during or after viral infection [7-9], their mechanisms of action have not been fully clarified. Recent evidence suggests that IP-10 is involved in RV-induced asthma exacerbation [16-18]. For example, RV infection induces bronchial epithelial cells to produce IP-10 *in vitro *and *in vivo *[16]. Monocytic cells also play an important role as a major source of IP-10 during RV infection [17,28]. Serum IP-10 concentrations are specifically increased in RV-induced asthma [18]. Furthermore, increased levels of IP-10 correlate with clinical disease severity during RV-induced exacerbation. These data suggest that IP-10 is likely to play a role in the pathogenesis of virus-induced asthma exacerbation.

Our results suggest that IP-10 induces eosinophil adhesiveness to ICAM-1, but not to VCAM-1 (Figure 1). Anti-CXCR3 Ab inhibited the induction of eosinophil adhesion (Figure 2B). Jinquan *et al. *reported that CXCR3 is expressed on eosinophils and that IP-10 induces chemotaxis [16]. Therefore, IP-10 could directly upregulate eosinophil functions via CXCR3. Indeed, in the present study, a combination of IP-10 and ICAM-1 activated eosinophil functions, such as O_2_^- ^generation (Figure 3A). Furthermore, IP-10 induced eosinophil degranulation (Figure 5) and the production of a number of cytokines/chemokines (Table 1). ICAM-1 is not only an adhesion molecule that plays an important role in inflammatory cell recruitment, but is also a cellular receptor for the majority (90%) of RVs [29]. Furthermore, RV infection increases ICAM-1 expression on epithelial cells [30,31]. Therefore, it is likely that eosinophil adhesion to epithelial cells through ICAM-1 would be enhanced during RV-induced asthma exacerbation. Moreover, eosinophil adhesion to ICAM-1 could activate eosinophil functions [11-13]. Since both IP-10 and ICAM-1 can be upregulated in RV-induced asthma exacerbation as described above, our results indicate that IP-10 interaction with ICAM-1 in the airway may play an important role in the activation and infiltration of eosinophils during RV-induced asthma exacerbation.

Not only CXCR3, but also other chemokine receptors are expressed on eosinophils. To date, constitutive and/or inducible expression of CCR1, CCR3, CXCR1, CXCR2, CXCR3, and CXCR4 has been reported in human eosinophils [15,32-34]. Of these receptors, high levels of CCR3 are constitutively expressed on eosinophils [35]. The CCR3 ligand, eotaxin, is a potent chemoattractant and activator of eosinophils, and thus the eotaxin/CCR3 axis presumably plays an essential role in the development of allergic inflammation. However, recent evidence indicates that chemokine receptor expression on eosinophils in peripheral blood differs from that on eosinophils in bronchoalveolar lavage fluid (BALF) [36-38], suggesting that chemokine receptors other than CCR3 also play important roles in eosinophil functions in the airway. For example, Liu *et al. *reported that eosinophils in the airway express more CXCR3 and less CCR3 than those in peripheral blood [36]. Katoh *et al. *found higher ratios of CXCR3-expressing eosinophils in BALF than in peripheral blood [37]. Nagase *et al. *reported that less CCR3 is expressed by eosinophils in BALF than in peripheral blood [38]. These findings suggest that eosinophils in the airways express more CXCR3 and less CCR3 than those in the peripheral circulation. The present study demonstrated that IP-10 activated the functions of eosinophils from peripheral blood. Therefore, CCR3 and CXCR3 might play similar roles in the activation and infiltration of eosinophils in the asthmatic airway. Since IP-10 is less sensitive to corticosteroid [18], we speculate that regulation of the CXCR3 ligand/CXCR3 axis would be important for regulating eosinophilic airway inflammation under specific conditions, such as viral infections.

In contrast to IP-10, the role of Mig or of I-TAC in the pathogenesis of asthma has not been fully clarified. Pillete *et al. *reported that segmental allergen challenge in asthmatic individuals induced a significant increase in IP-10, but not in Mig or I-TAC, in BALF [39]. Fulkerson *et al. *reported that Mig inhibits eosinophil responses to diverse stimuli [40], suggesting an immune suppressive role of Mig in some circumstances. The present study demonstrated that both Mig and I-TAC upregulate eosinophil functions such as adhesion to rh-ICAM-1 and O_2_^- ^generation (Figure 4). Moreover, Mig and I-TAC-enhanced eosinophil adhesion and respiratory burst were suppressed by anti-CXCR3 Ab. These results suggested that both Mig and I-TAC could directly activate eosinophils through CXCR3. However, neither Mig nor I-TAC modified the release of EDN by eosinophils, and their effect on eosinophil adhesion or cytokine/chemokine production was weaker than that of IP-10 (Figures 4 and 5, and Table 1). Therefore, Mig or I-TAC might have lesser significance for the pathogenesis of eosinophilic airway inflammation than IP-10.

## Conclusions

In conclusion, our results indicate that CXCR3 ligands upregulate eosinophil functions such as adhesion and O_2_^- ^generation through CXCR3 and β_2 _integrin. Furthermore, IP-10 increased eosinophil release of EDN and eosinophil production of a number of cytokines/chemokines. Therefore, inhibition of the CXCR3 ligand/CXCR3 axis might serve as a strategy to suppress eosinophilic airway inflammation, especially in virus-induced asthma exacerbation.

## List of abbreviations

ANOVA: analysis of variance; BALF: bronchoalveolar lavage fluid; cysLT: cysteinyl leukotriene; EDN: eosinophil-derived neurotoxin; EPO: eosinophil peroxidase; FCS: fetal calf serum; HBSS: Hank's balanced salt solution; ICAM: intercellular adhesion molecule; IP-10: IFN-γ-inducible protein of 10 kDa; I-TAC: IFN-inducible T cell α chemoattractant; LT: leukotriene; Mig: monokine induced by IFN-γ; O_2_^-^: superoxide anion; RV: rhinovirus; SOD: superoxide dismutase; VCAM: vascular cell adhesion molecule.

## Competing interests

The authors declare that they have no competing interests.

## Authors' contributions

YT carried out the experiments, analyzed the data, and drafted the manuscript. KN participated in direction of the study, analyzed the data, and wrote the manuscript. TK carried out the eosinophil experiments. KH and MK participated in the data analyses. MN participated in direction of the study and edited the manuscript. All authors read and approved the final manuscript.
